# The Prevalence of Shiga Toxin-Producing *Escherichia coli* and Enteropathogenic *Escherichia coli* Isolated from Raw Chicken Meat Samples

**DOI:** 10.1155/2021/3333240

**Published:** 2021-12-27

**Authors:** Omid Zarei, Leili Shokoohizadeh, Hadi Hossainpour, Mohammad Yousef Alikhani

**Affiliations:** ^1^Student Research Committee, Hamadan University of Medical Sciences, Hamadan, Iran; ^2^Department of Microbiology, Faculty of Medicine, Hamadan University of Medical Sciences, Hamadan, Iran

## Abstract

**Background:**

Shiga toxin-producing *Escherichia coli* (STEC) is known as a crucial zoonotic food-borne pathogen. A total of 257 raw chicken meat samples were collected from different markets in Hamadan, west of Iran, from January 2016 to May 2017.

**Materials and Methods:**

The samples were cultured in selective and differential culture media, and the virulence genes of *E. coli* isolates were analyzed by PCR assay. The antibiotic resistance patterns of *E. coli* isolates were determined by the disk diffusion method. The genetic relatedness of the *E. coli* O157 isolates was analyzed by ERIC-PCR.

**Results:**

In total, 93 (36% ± 3.12) of the isolates were identified as *E. coli* in this study. Based on serological and microbiological tests, 36 (38.7% ± 9.9), 7 (7.5% ± 5.35), and 12 (12.9% ± 6.81) of the *E. coli* isolates were characterized as STEC, enteropathogenic *E. coli* (EPEC), and attaching and effacing *E. coli* (AEEC) strains, respectively. A high level of resistance to nalidixic acid (91.4% ± 5.7), tetracycline (89.2% ± 6.31), ampicillin (82.8% ± 7.67), and trimotoprime-sulfametoxazole (71% ± 9.22) was detected among the *E. coli* isolates. The analysis of the ERIC-PCR results showed five different ERIC types among the *E. coli* O157 isolates.

**Conclusions:**

Based on our findings, control and check-up of poultry meats should be considered as a crucial issue for public health.

## 1. Introduction

Enterobacteriaceae family bacteria are one of the most common Gram-negative bacilli, some of which are normal flora and some are pathogenic. Diarrheagenic *E. coli*, which causes diarrhea in humans, can be classified into seven different pathotypes on the basis of its specific virulence properties, distinct epidemiology, and clinical features: Enterotoxigenic *E. coli* (ETEC), Enteroinvasive *E. coli* (EIEC), Enteroaggregative *E. coli* (EAEC), Diffuse-Adhering *E. coli* (DAEC), Enteropathogenic *E. coli* (EPEC), and Shiga toxin-producing *E. coli* (STEC) [1]. STECs are one of the most important pathogens transmitted by food. In addition to causing food poisoning, these strains can cause severe diseases such as diarrhea, bleeding colitis, hemolytic uremic syndrome, thrombocytopenic purpura, and death. Most cases of ulcerative colitis and hemolytic uremic syndrome are related to the O157:H7 serotype which is considered as the most important serotype of this strain. Several outbreaks of bacterial food-borne disease due to the consumption of undercooked or raw meat contaminated with STEC strains have been reported [1, 2].

In addition to Shiga toxins, an external membrane protein called intimin is responsible for the attachment of bacteria to the intestinal epithelial cells, causes a certain damage (called “attaching-effacing (A/E) lesions”), and is encoded by the *eae* gene [3, 4]. Also, enterohemolysin, encoded by the *hly* gene, is an effective factor in the pathogenicity of STEC [5]. Because only limited and incomplete studies have been conducted on the prevalence and epidemiology of the O157 : H7 serotype in developing countries, its prevalence has been reported as low [6].

The EPEC pathovar plays an important role as a causative agent of infantile diarrhea in developing countries [7]. This pathovar has intimin which is encoded by the chromosomal *eae* gene. It also possesses the ability to form A/E lesions on intestinal cells but does not contain Shiga toxin-encoding genes.

Attaching and effacing *E. coli* (AEEC) are characterized by their ability to cause attaching and effacing (A/E) lesions in the gut mucosa of human and animal hosts leading to diarrhea. Thus, two groups of *Escherichia coli* strains (STEC and EPEC) that cause attaching and effacing (A/E) lesions are classified as AEEC. In Iran, most molecular studies on the STEC pathovar have been conducted on dairy and animal stool samples, and little information is available on STEC and EPEC strains from poultry sources. Therefore, the aim of this study was to detect the virulence factors *stx1*, *stx2*, *eae*, and *hlyA* in the *E. coli* isolates and also to perform molecular typing of O157 : H7 strains isolated from a raw chicken meat sample.

## 2. Materials and Methods

### 2.1. Phenotypic Identification of *Escherichia coli* Strains

In this cross-sectional study, a total of 257 raw chicken meat samples were randomly collected by using an electronic random number generator (https://www.randomresult.com) from different butchers and supermarkets of different areas of Hamadan city, west of Iran, from January 2016 to May 2017. The present study was ethically approved by the Institutional Review Board of Hamadan University of Medical Sciences (IR.UMSHA.REC.1397.444). The meat samples were transferred to sterile tubes, were transported to tubes containing thioglycolate broth media after homogenization, and were incubated overnight at 37°C. Then, they were inoculated on MacConkey agar plates (Merck, Germany) and incubated at 37°C for 24 h. Subsequently, the *E. coli*-like colonies were subjected to different biochemical tests including sugar fermentation, Simmons' citrate, indole production, motility, methyl red, and Voges–Proskauer (IMVIC) tests [8]. The sorbitol MacConkey agar (Merck, Germany) and sero grouping with anti-O157 sera (Baharafshan, Iran) were used for the diagnosis of the *E. coli* O157 serotypes.

### 2.2. Molecular Detection of *Escherichia coli* Pathotypes

A sweep of five *E. coli* colonies on the MacConkey agar was inoculated in LB broth and incubated overnight at 37°C, and the genomic DNAs of the colonies were extracted by the boiling method [9]. For detection of *E. coli* pathotypes, the virulence genes s*tx1*, *stx2*, *hlyA*, and *eae* were detected by the PCR method using the primers described in previous studies [10].

The virulence genes *eae*, *stx1*, and *stx2* were detected using a triplex PCR in a reaction mixture with a total volume of 20 *μ*L, PCR Master Mix 2x (Fermentas, Lithuania) of 10 *μ*L, 6 *μ*L sterile double distilled water, 1 *μ*L from each primer (10 pmol/*µ*l), and 2 *μ*L DNA template. The cycling program was used as follows: initial denaturation (3 min at 94°C), followed by 35 cycles of denaturation (1 min at 94°C), annealing (1 min at 55°C), extension (1 min at 72°C), and final extension (7 min at 72°C). For the *hly* gene, a single PCR reaction was performed with the same conditions as mentioned above except that annealing was at 63°C for 1 min. The PCR products were detected by staining with SYBR Safe and gel electrophoresis on 1% agarose gels and, finally, were visualized under UV light.

### 2.3. Antimicrobial Susceptibility Testing

The antimicrobial susceptibility of *E. coli* isolates to cefotaxime (CTX), ceftazidime (CAZ), cefepime (CPM), cefoxitin (FOX), cefexime (CFM), nitrofurantoin (NIT), gentamicin (GM), ciprofloxacin (CIP), nalidixic acid (NA), trimethoprim-sulfamethoxazole (SXT), aztreonam (ATM), amoxicillin (A), ampicillin (AMP), tetracycline (TET), minocycline (MIN), and imipenem (IPM) was detected by the disk diffusion method according to CLSI guidelines [11].

### 2.4. ERIC-PCR of *E. coli* O157 Serotype Isolates

For molecular typing and detection of the genetic linkage among *E. coli* O157 serotype strains, ERIC-PCR was carried out using ERIC primers and the conditions described in a previous study [12]. The banding patterns of ERIC were analyzed by an online data analysis service (inslico.ehu.es). The ERIC profiles were compared by the Dice method and were clustered by the UPGMA program.

## 3. Statistical Analysis

This study was a descriptive-application study. SPSS V11 software was used for the statistical analyses. The *P* value and confidence intervals were less than 0.05 and 95%, respectively.

## 4. Results

Among the 257 raw poultry samples, 93 (36% ± 3.12) isolates were identified as *E. coli*. Based on serological and microbiological tests, 36 (38.7% ± 9.9), 7(7.5% ± 5.35), and 12 (12.9% ± 6.81) of the *E. coli* isolates were characterized as STEC (*stx*1^+^ and/or *stx*2^+^and *eae+*/*eae*^*_*^), EPEC (positive for *eae*), and AEEC (EPECs and *eae* + strains of STECs) strains, respectively. All of the STEC isolates showed colorless colonies on the MacConkey sorbitol media culture.

**Figure 1:**
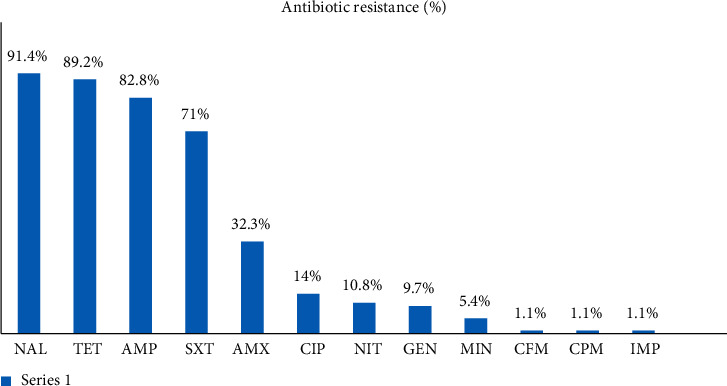
Antibiotic resistance (%) of *E. coli* isolated from raw chicken meat.

The results of the antimicrobial susceptibility test conducted on 93 *E. coli* isolates are shown in Figure 1. Based on the results of the antimicrobial susceptibility test, all of the isolates were susceptible to cefotaxime, cefoxitin, ceftazidime, and aztreonam. A high level of resistance to nalidixic acid (91.4 ± 5.7%), tetracycline (89.2% ± 6.31), ampicillin (82.8% ± 7.67), and trimethoprim-sulfamethoxazole (71% ± 9.22) was detected among the *E. coli* isolates.

**Figure 2:**
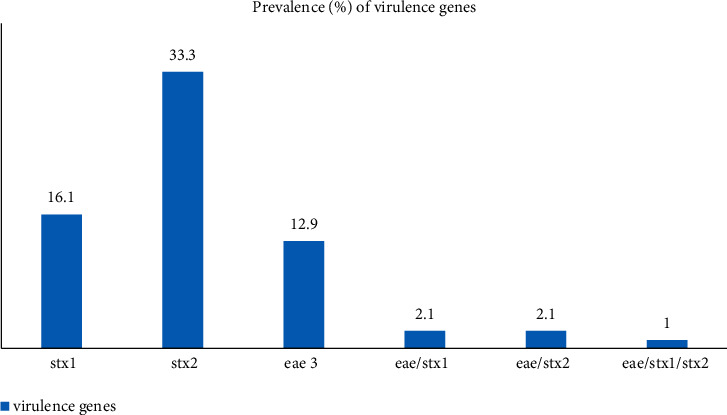
Prevalence of virulence genes among 93 *E. coli* isolates isolated from raw chicken meat.

**Figure 3:**
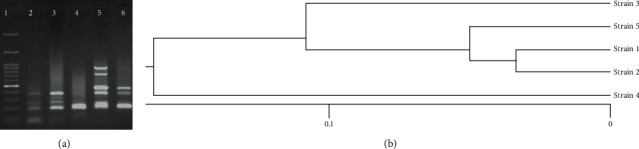
(a): Different ERIC patterns of 5 different *E. coli* O157 isolates; 1: ladder (100 bp), lane numbers 2–6: *E. coli* O157; (b) dendrogram of ERIC-PCR analysis.

The PCR results showed that the distribution of the virulence genes *stx*1, *stx*2, and *eae* among the 93 *E. coli* isolates was 15(16.1 ± 7.47%), 31(33.3% ± 9.58), and 12(12.9% ± 6.81), respectively. All of *E. coli* O157 strains showed *stx1*^*+*^*/stx2*^*+*^*/eae*^*+*^ (1 isolate), *stx1*^*+*^*/eae*^*+*^ (2 isolates), and *sx2*^*+*^*/eae*^*+*^ (2 isolates) patterns. The *hly*A gene was not detected in any of the *E. coli* isolates (Figure 2). The analysis of the ERIC-PCR results showed genetic diversity among *E. coli* O157 strains because five different ERIC patterns were observed among these strains (Figure 3).

## 5. Discussion

The results of our study have shown that chicken meat can be contaminated with *E. coli*. The *stx*2 gene was the most frequent virulence factor among the STEC isolates. The major animal source of STEC is primarily cattle, followed by sheep, goats, pigs, and poultry. Poultry meat is known as the potential source of STEC contamination compared to other sources of meat. In Korea, STEC was isolated in 22.6% of beef, 7.3% of poultry, and 2.0% of pork meat samples [13].

In the current study, O157 *E. coli* isolates were detected in 5.3% of the poultry meat samples and recognized as STEC strains. Although the prevalence of this isolate was not significant, this rate of infection is considerable from the public health point of view. The prevalence of STEC and AEEC in the current study is different from that in some studies in Iran and other countries. In the current study, higher STEC and lower AEEC isolates were detected compared to the study of Momtaz and Jamshidi [14]. They reported that the prevalence of STEC and AEEC was 21% and 34%, respectively, among 422 raw poultry meat samples in different cities of Iran [14]. They also reported that *stx1* was the most frequent (96%) virulence factor among the isolates. In contrast, in the current study, *stx1* was found only in 16% of the isolates. One of the reasons for this difference in frequency can be the difference in the number of samples studied. However, Guran et al. showed that the overall prevalence of *E. coli* O157 in poultry meat samples collected from supermarkets in Diyarbakir, Turkey, was 1.3% [15]. One of the significant results of the current study is that 12.9% of the *E. coli* isolates were identified as AEEC. Intimin genes are present in EPEC and in some STEC. Atypical enteropathogenic *E. coli* (EPEC) or AEEC appear to be more closely related to STEC [16–19]. Based on the results of the current study, the role of AEEC strains in gastrointestinal infection needs further investigations.

In this study, the *E. coli* O157 strains were positive for *stx1*, *stx2*, and *eae* genes. In India, Dutta et al. reported that 14 (33.33%) isolates carried at least 1 virulence gene. Ten (23.81%) of these isolates (collected from poultry samples) were recorded as STEC, and 4 (9.52%) of them were recorded as EPEC [20].

Some gastrointestinal infections caused by *E. coli* have a bacterial origin and need to be treated by antibiotics. In a review study by Roth et al., the resistance rates of *E. coli* strains (isolated from broiler samples) to tetracycline, trimethoprim-sulfamethoxazole, streptomycin, and ampicillin were more than 40% in all the studied countries. Increasing antibiotic resistance is a major concern for animal and human health because of the high consumption of antibiotics in veterinary medicine. Resistant bacteria can spread from food-producing animals to humans. The information from the evaluated countries indicates that antibiotics such as tetracycline, aminoglycoside, sulfonamide, and penicillin are usually used in the poultry industry [21].

In this study, the resistance levels of STEC to some antimicrobial agents such as nalidixic acid, ampicillin, tetracycline, and trimethoprim-sulfamethoxazole ranged from 71 to 91%. According to these results, the poultry meat contaminated with STEC strains can be a potential source of antimicrobial resistance. In a study conducted in Thailand, 100% of the *E. coli* isolates showed resistance to tetracycline, ampicillin, and erythromycin.

Momtaz and Jamshidi reported the high resistance of STEC strains to tetracycline, chloramphenicol, and nitrofurantoin (63 to 77%). According to our findings and studies by others, the prescription of tetracycline is recommended neither in cases of *E. coli* infection nor in veterinary medicine with respect to poultry products [14].

There are few reports about the molecular typing of STECs from poultry sources in Iran and other countries. In the current study, ERIC-PCR genotyping demonstrated 5 different ERIC-genotypes from 5 *E. coli* O157 isolates. Therefore, the results of the current study showed genetic diversity among *E. coli* O157 isolates as well as the different potential sources of *E. coli* O157 contamination. The results of the present research also indicated the usefulness of the PCR-based genotyping method in the epidemiological investigations of virulent *E. coli* strains and of STEC strains. There are some limitations in this study due to the lack of access to human samples because finding relevant human samples is very difficult except during infection outbreaks. Consistent with our results, in a study by Shekar et al. in India, the ERIC-PCR results discriminated 12 STEC isolates from poultry samples into 11 ERIC-PCR genotypes [22].

## 6. Conclusions

The results of the current study revealed that poultry meat can be considered as a source of pathogenic *E. coli* strains. Pathogenic *E. coli* strains in poultry meat samples were detected by accurate and quick techniques such as PCR assay, and the detection of STEC (38%) was a significant finding. Our results highlight the need to pay more attention to controlling poultry meat and also antibiotic prescription in veterinaries. Our results indicated that the *E. coli* virulence genes, especially *stx1, stx2,* and *eae*, existed to a large extent in pathogenic *E. coli* strains isolated from poultry meat.

## Data Availability

The data used to support the findings of this study are included within the article.

## References

[1] Bouzari S., Farhang E., Hosseini S. M., Alikhani M. Y. (2018). Prevalence and antimicrobial resistance of shiga toxin-producing *Escherichia coli* and enteropathogenic *Escherichia coli* isolated from patients with acute diarrhea. *Iranian Journal of Microbiology*.

[2] Dehkordi F. S., Parsaei P., Saberian S. (2012). Prevalence study of theileria annulata by comparison of four diagnostic T. *Bulgarian Journal of Veterinary Medicine*.

[3] Momtaz H., Davood Rahimian M., Safarpoor Dehkordi F. (2013). Identification and characterization of *Yersinia enterocolitica* isolated from raw chicken meat based on molecular and biological techniques. *The Journal of Applied Poultry Research*.

[4] Exeni R. A., Fernandez-Brando R. J., Santiago A. P. (2018). Pathogenic role of inflammatory response during shiga toxin-associated hemolytic uremic syndrome (HUS). *Pediatric Nephrology*.

[5] Smith J. L., Fratamico P. M. (2017). *Escherichia coli* as a pathogen. *Foodborne Diseases*.

[6] Pasquali F., Palma F., Trevisani M. (2018). Whole genome sequencing based typing and characterisation of shiga-toxin producing *Escherichia coli* strains belonging to O157 and O26 serotypes and isolated in dairy farms. *Italian Journal of Food Safety*.

[7] Parreira V. R., Arns C. W., Yano T. (1998). Virulence factors of avian *Escherichia coli* associated with swollen head syndrome. *Avian Pathology*.

[8] Aslani M., Alikhani M. (2009). Serotypes of enteropathogenic *Escherichia coli* isolated from children under 5 years of age. *Iranian Journal of Public Health*.

[9] Wayne P. (2011). *Performance Standards for Antimicrobial Susceptibility Testing: Twenty-First Informational Supplement: CLSI Document M100-S21*.

[10] Zamani A., Yousefi Mashouf R., Ebrahimzadeh Namvar A. M., Alikhani M. Y. (2013). Detection of magA gene in klebsiella spp. isolated from clinical samples detection of magA. *Iranian Journal of Basic Medical Sciences*.

[11] Fagan P. K., Hornitzky M. A., Bettelheim K. A., Djordjevic S. P. (1999). Detection of shiga-like toxin ( stx 1 and stx 2 ), intimin ( eaeA ), and enterohemorrhagic *Escherichia coli* (EHEC) hemolysin (EHEC hlyA ) genes in animal feces by multiplex PCR. *Applied and Environmental Microbiology*.

[12] Zarei O., Shokoohizadeh L., Hossainpour H., Alikhani M. Y. (2018). Molecular analysis of *Pseudomonas aeruginosa* isolated from clinical, environmental and cockroach sources by ERIC-PCR. *BMC Research Notes*.

[13] Lee G. Y., Jang H. I., Hwang I. G., Rhee M. S. (2009). Prevalence and classification of pathogenic *Escherichia coli* isolated from fresh beef, poultry, and pork in Korea. *International Journal of Food Microbiology*.

[14] Momtaz H., Jamshidi A. (2013). Shiga toxin-producing *Escherichia coli* isolated from chicken meat in Iran: serogroups, virulence factors, and antimicrobial resistance properties. *Poultry Science*.

[15] Guran H. S., Vural A., Erkan M. E., Durmusoglu H. (2017). Prevalence and some virulence genes of *Escherichia coli* O157 isolated from chicken meats and giblets. *Annals of Animal Science*.

[16] Fröhlicher E., Krause G., Zweifel C., Beutin L., Stephan R. (2008). Characterization of attaching and effacing *Escherichia coli* (AEEC) isolated from pigs and sheep. *BMC Microbiology*.

[17] Trabulsi L. R., Keller R., Gomes T. A. T. (2002). Typical and atypical enteropathogenic *Escherichia coli*. *Emerging Infectious Diseases*.

[18] Beutin L., Marchés O., Bettelheim K. A. (2003). HEp-2 cell adherence, actin aggregation, and intimin types of attaching and effacing *Escherichia coli* strains isolated from healthy infants in Germany and Australia. *Infection and Immunity*.

[19] Ferens W. A., Hovde C. J. (2011). *Escherichia coli* O157 : H7: animal reservoir and sources of human infection. *Foodborne Pathogens and Disease*.

[20] Dutta T. K., Roychoudhury P., Bandyopadhyay S., Wani S. A., Hussain I. (2011). Detection & characterization of shiga toxin producing *Escherichia coli* (STEC) & enteropathogenic *Escherichia coli* (EPEC) in poultry birds with diarrhoea. *Indian Journal of Medical Research*.

[21] Roth N., Käsbohrer A., Mayrhofer S., Zitz U., Hofacre C., Domig K. J. (2019). The application of antibiotics in broiler production and the resulting antibiotic resistance in *Escherichia coli*: a global overview. *Poultry Science*.

[22] Sekhar M. S., Sharif N. M., Rao T. S., Metta M. (2017). Genotyping of virulent *Escherichia coli* obtained from poultry and poultry farm workers using enterobacterial repetitive intergenic consensus-polymerase chain reaction. *Veterinary World*.

[23] Shokoohizadeh L., Hossainpour H., Alikhani M. Y. (2019). Prevalence of shiga toxin-producing *Escherichia coli* isolated from chicken meat in west of Iran. https://www.researchsquare.com/article/rs-2323/v2.

